# Formation Mechanisms for Phosphorene and SnIP

**DOI:** 10.1002/anie.202016257

**Published:** 2021-02-15

**Authors:** Markus R. P. Pielmeier, Tom Nilges

**Affiliations:** ^1^ Department of Chemistry Technical University of Munich (TUM) Lichtenbergstrasse 4 85748 Garching b. München Germany

**Keywords:** ab initio calculations, black phosphorus/phosphorene, materials science, reaction mechanism, SnIP double helix material

## Abstract

Phosphorene—the monolayered material of the element allotrope black phosphorus (P_black_)—and SnIP are 2D and 1D semiconductors with intriguing physical properties. P_black_ and SnIP have in common that they can be synthesized via short way transport or mineralization using tin, tin(IV) iodide and amorphous red phosphorus. This top‐down approach is the most important access route to phosphorene. The two preparation routes are closely connected and differ mainly in reaction temperature and molar ratios of starting materials. Many speculative intermediates or activator side phases have been postulated especially for top‐down P_black_/phosphorene synthesis, such as Hittorf's phosphorus or Sn_24_P_19.3_I_8_ clathrate. The importance of phosphorus‐based 2D and 1D materials for energy conversion, storage, and catalysis inspired us to elucidate the formation mechanisms of these two compounds. Herein, we report on the reaction mechanisms of P_black_/phosphorene and SnIP from P_4_ and SnI_2_ via direct gas phase formation.

## Graphical abstract

1D‐SnIP and 2D‐phosphorene are formed in a gas phase reaction of gaseous white phosphorus (P_4_) and SnI_2_ via P_4_ activation followed by successive helix or layer growth. P_4_ activation takes place by Sn_2_I_4_ dimers attracting the P lone pairs (SnIP) or direct SnI_2_ insertion in a P−P bond (phosphorene). Both reaction mechanisms were studied between 0 K and synthesis temperature.

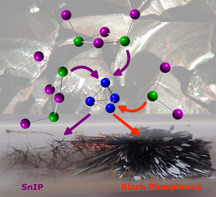

## Introduction

For many years the research on phosphorous allotropes and compounds has led to fascinating results and has not yet come to an end.^[1]^ Allotropes like red phosphorous have been known for many decades, but they are still controversially discussed in the community while trying to unveil their structure with solid data.^[2]^

Phosphorene is an important 2D material with many possible applications in modern technology.^[3]^ Fields of application are sensors,^[4]^ field effect transistors (FETs),^[5]^ catalysts for water splitting^[6]^ or hydrogen evolution, and active materials for batteries.^[7]^ Mono‐ and multilayer phosphorus materials are investigated by quantum chemists and physicists owing to their conformational flexibility^[8]^ (as single sheet), stacking variability and Moiré‐driven optical transitions (double and multiple layers).^[9]^ Recently, an extensive theoretical work was published discussing possible conformations of a single phosphorene sheet, which consist of two chair conformers, a black phosphorus monolayer, a gray arsenic‐type (rhombohedral phosphorus or blue phosphorus) and a boat conformer.^[8]^ Phosphorene is often prepared by a top‐down approach from black phosphorus (P_black_), which defines the P_black_ synthesis as a crucial step in phosphorene science. The most feasible top‐down approach to phosphorene is delamination of large P_black_ crystals made by the aforementioned synthesis process. In the past few years, bottom‐up approaches have emerged and several studies have been performed to identify a possible reaction mechanism for this P_red_‐to‐P_black_ gas‐phase transformation reaction in CVD processes.[^10a^, ^10b^] In solution, another bottom‐up reaction mechanism has been evaluated and investigated, featuring a nucleophilic attack of a free lone pair of ethylenediamine (en) on P_white_ (P_4_ molecule)^[7a]^ and P_red_.^[10c]^ Here, the P_4_ entity (P_white_) is opened to a reactive species, which tends to rearrange into layers of corrugated P_black_ sheets afterwards. Unfortunately, this solution‐based synthesis with ethylenediamine leads to P−N bond formation and nitrogen impurities in P_black_, which has been proven by XPS.^[10c]^ Such impurities, which form in addition to common oxygen impurities caused by oxidation processes in solution and during workup procedures, can affect the performance of P_black_ in applications. Using a gas‐phase‐based synthesis route, the solvent influence can fully be suppressed and the oxidation problem can be minimized. Also, the crystal quality is generally much higher than for solution‐based processes. Therefore, the gas‐phase‐based synthesis remains the crucial method to grow pure, highly crystalline, large‐area crystals of P_black_ for a top‐down fabrication of phosphorene.

**Figure 1:**
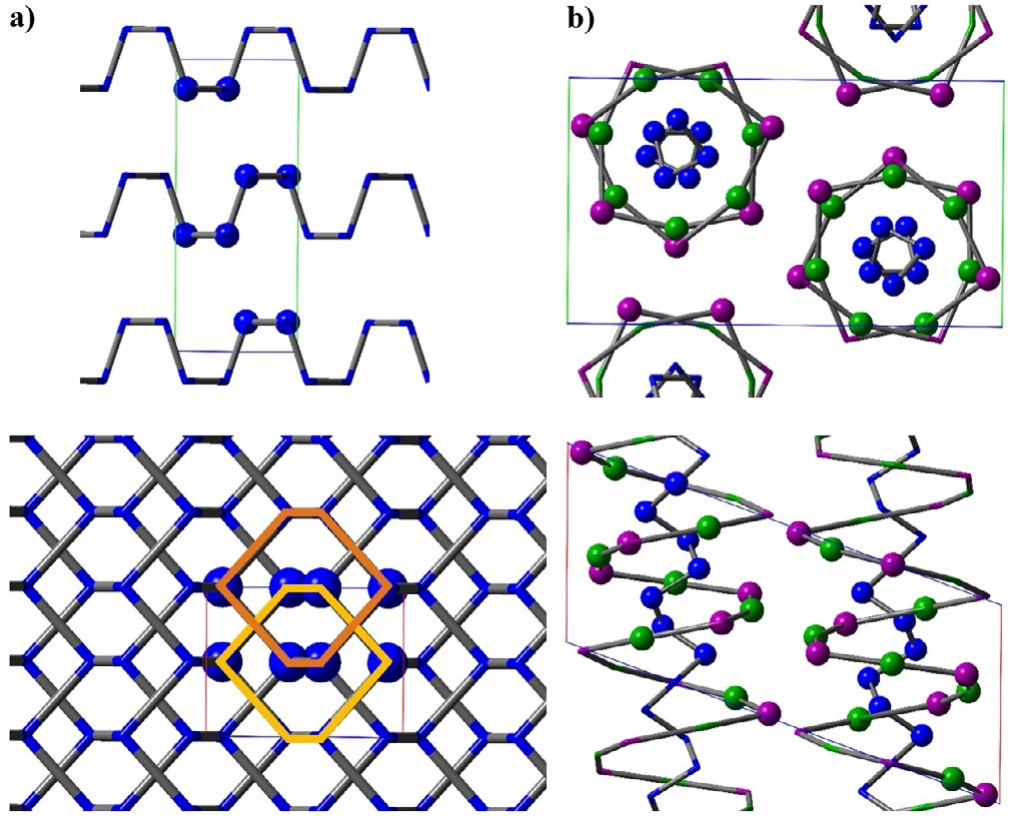
Crystal structure sections of a) orthorhombic P_black_ and b) double helical SnIP. Top row, projections drawn along the *a*‐axis and bottom row along the *b*‐axis. Atoms in unit cells are shown as balls, while others are depicted as sticks. Orange hexagons outline AB stacking order for two visible planes.

SnIP, an inorganic double helix material, consists of an outer [SnI]^+^ and an inner [P]^−^ helix.^[11]^ Each chiral SnIP double helical strand has a diameter of approx. 1 nm and shows either left‐ (*M* helix) or right‐handed (*P* helix) chirality (Figure 1). Those two different enantiomeric rods appear as a racemic mixture arranged in a pseudo‐hexagonal rod‐packing along the *a* axis. After SnIP′s discovery the preparation process was optimized by varying, for example, temperature profile and reactant ratios, and the stereochemistry as well as stacking variations of chiral double helices in SnIP bulk material were investigated.^[12]^

Herein, our investigation focuses on the formation mechanisms of the two title compounds. Taking the state‐of‐the‐art knowledge on P_black_ synthesis and its formation into account, as nicely reviewed by Wang et al., it becomes obvious that the formation mechanism is still unknown.^[1h]^ Several compounds, such as other phosphorus allotropes or ternary phases (Sn_24_P_19.3_I_8_),^[11c]^ are postulated as intermediates in the formation process.^[1h]^ It has been shown in an in situ experiment that P_black_ grows within minutes at temperatures significantly below synthesis temperatures reported earlier and without side phase formation.^[3b]^ In general, such fast formation of P_black_ is very unlikely if intermediates need to be formed first to allow epitaxial growth on it (e.g. clathrate‐type Sn_24_P_19.3_I_8_) afterwards. The mere existence of side phases even in close vicinity to the desired product does not necessarily indicate an epitaxial growth mechanism. It has been shown earlier that SnIP decomposes peritectically to the aforementioned clathrate^[11b]^ and it is therefore possible that SnIP may itself act as a precursor to form an intermediate clathrate or P_black_ directly. This speculation is at least feasible and might be another possible reaction pathway to be evaluated. An Sn‐I‐P intermediate was postulated first in 2016 in the literature^[11b]^ and was discussed thereafter in other publications.^[13]^ In none of the publications a clearly defined species has been identified which may explain the formation of P_black_ from simple materials via the gas phase. To stop speculation, we started a DFT‐based investigation to envision formation mechanisms for SnIP at lower and P_black_ at higher temperatures.

## Results and Discussion

We applied density functional theory (DFT) to unveil the gas phase formation process of two intriguing materials: P_black_, an element allotrope of phosphorus, and SnIP, the first inorganic atomic‐scale double helix compound (cf. Figure 1). P_black_ represents a precursor to 2D phosphorene, and SnIP is a quasi‐1D material consisting of weakly bonded SnIP double helices. Within the framework of DFT we generated and optimized all local minimum structures along the reaction pathways using the Gaussian09 code.^[14]^ Ab initio calculations were performed on GGA level and with the PBE functional. All relevant DFT data are summarized in the Computational Methods Section in the Supporting Information. The main gas phase species were derived from **Cal**culated **Pha**se **D**iagram (CalPhaD) methods and thermodynamic data were calculated with the tragmin5.1 program suite.^[15]^ In the following, we investigated the energy landscape at different temperatures and found detailed, chemically reasonable and energetically feasible reaction pathways capable of explaining all experimental observations during synthesis. We explored many probable combinations and summarize the most promising pathways in this work.

Despite obvious structural differences, the synthesis procedures for both compounds are rather similar. In both cases tin, Sn^IV^ iodide, and amorphous red phosphorus (P_red_) act as starting materials.^[16]^ While catalytic amounts of Sn and SnI_4_ are necessary to transform P_red_ into P_black_, equimolar ratios are needed for SnIP. A second important difference is the synthesis temperature window. P_black_ can be synthesized from 923 to 823 K, while SnIP is formed below 673 K. For further details concerning synthesis procedures and structural details, see the Supporting Information.

A detailed mechanism for the gas‐phase‐based P_black_ synthesis, which includes a quantum chemical investigation with local minima structures along the reaction pathway or alternatively the identification of any intermediate along the reaction pathway, is lacking. So far, only assumptions, such as the occurrence of epitaxial growth of P_black_ on Sn_24_Sn_19.3_I_8_ or the formation of other phosphorus allotropes as precursor states, are used to explain or interpret the formation of P_black_.

**Figure 2:**
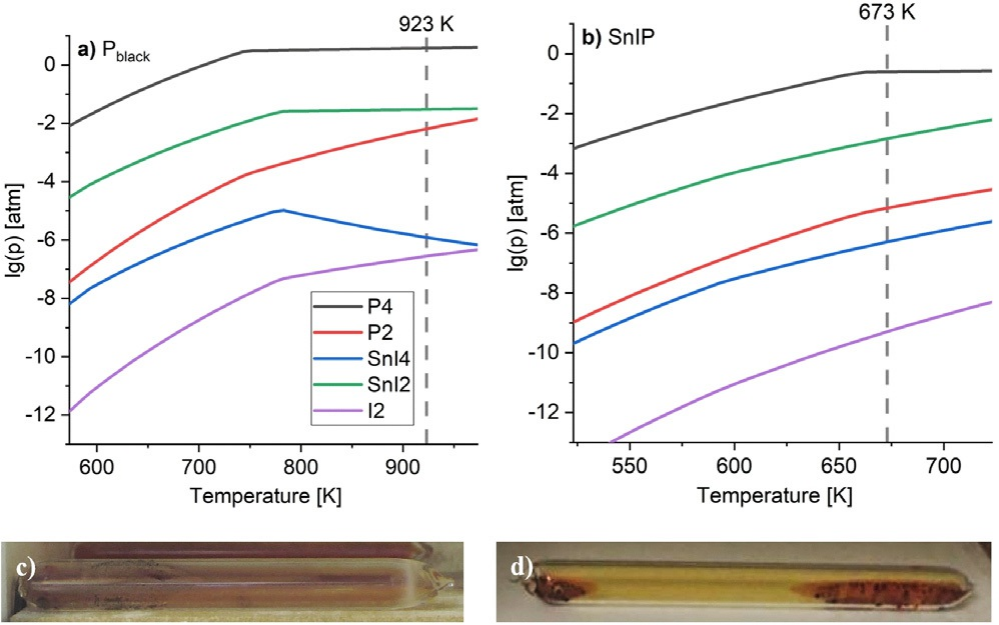
a) Partial pressure diagram featuring the corresponding equilibrium gas phase pressure during P_black_ synthesis in the temperature range from 600 to 950 K. b) Partial pressure diagram for SnIP from 500 to 750 K. Partial pressures are plotted on a logarithmic scale. The dashed lines show the maximum synthesis temperature (*T*_max_) applied for each case. Silica glass ampoules in the oven during synthesis at c) *T*_max_=923 K (P_black_) and d) *T*_max_=673 K (SnIP). Sn (boiling point approx. 2600 °C) has no significant partial pressure in the given temperature range.

In a first evaluation step, we calculated the gas phase species to occur in the Sn/SnI_4_‐based synthesis of P_black_ and SnIP by CalPhaD methods. The latter is a powerful tool to investigate gas phase transport reactions.^[17]^ We repeated CalPhaD calculations for P_black_, substantiating the results reported by Lange et al. in 2007 (see Figure 2 a),^[16]^ and derived new ones for SnIP (Figure 2 b). In both cases the dominating gas phase species with significant partial pressures are P_4_ (**1**) and SnI_2_ (**2**), while SnI_4_ (**3**), I_2_ (**4**), or P_2_ does not exhibit mentionable presence. SnI_2_ (green curves) and P_4_ (black curves) partial pressures (Figure 2) are at least 3 orders of magnitude higher than any competing molecules in equilibrium. I_2_ partial pressures are very low in both cases, even in the SnIP synthesis, where significant (equimolar) amounts of SnI_4_ (**3**) are present, which possibly acts as an iodide source. It is therefore likely that the two main components **1** and **2** play a crucial role in the formation of both compounds. The decisive role of **1** was already proven in the phosphorene synthesis by Köpf in 2014 by in situ experiments.^[3b]^ In all cases we reach saturation vapor pressures, represented by almost horizontal curves for **1** and **2** in Figure 2, prior to the applied maximum reaction temperatures (illustrated by dashed lines in Figure 2). During P_black_ synthesis (which starts at 923 K and continues during cooling) the P_4_ saturation pressure remains present over a long temperature range from 923 to approx. 740 K. This feature is beneficial for the fast growth of P_black_, which occurs within minutes in a temperature range from 773 to 673 K.^[3b]^

In order to verify the results of our CalPhaD calculations we investigated the ampoules under synthesis conditions by opening the ovens at its respective reaction temperatures (see Figure 2 c,d; videos given in Supporting Information Note 1). We observed a light orange gas atmosphere and condensation of **2** during P_black_ synthesis (**2** shows an evaporation rate of 127 mg h^−1^ at 623 K and ambient pressure^[18]^) but no hints for **4** at both reaction temperatures. **4** is obviously not present and can therefore not contribute to any reaction mechanism. In gas phase balance and mass spectrometry experiments during P_black_ and As_0.83_P_0.17,black_ experiments only metal diiodide, Pn_4_, and Pn_2_ fragments (Pn=P, As) were identified as main gas phase species.[^1k^, ^16^]

**Figure 3:**
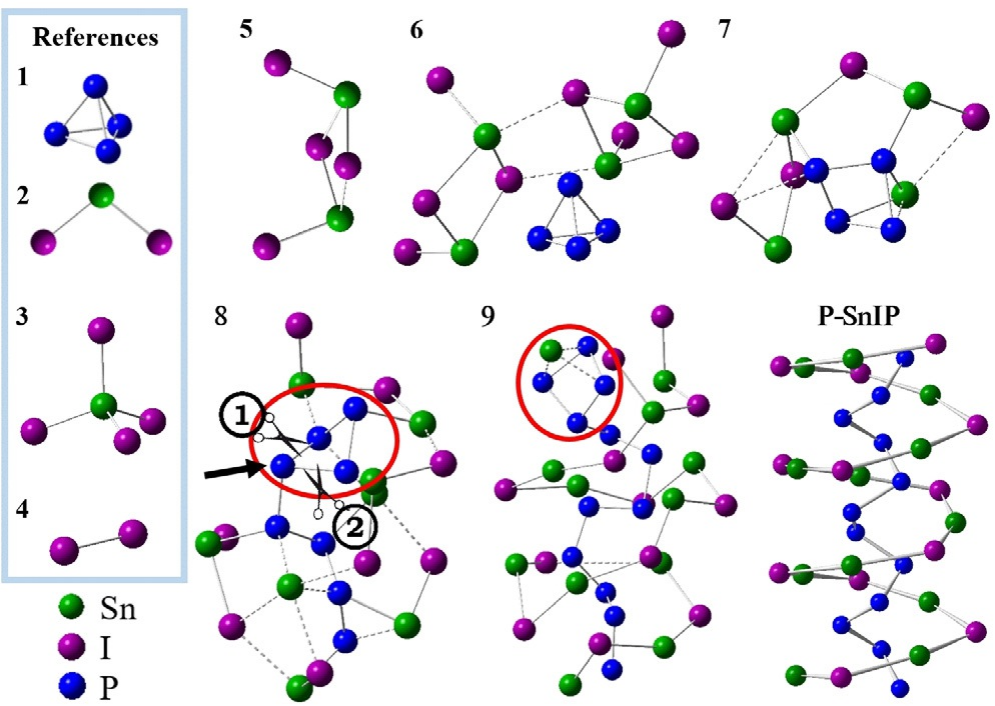
Local minimum structures along the reaction pathway to SnIP. Important starting materials (**1**, **2**) and a Sn_2_I_4_ dimer **5** are necessary to initiate SnIP formation. **3** and **4** are neglected due to chemical considerations and observations. P_4_ activation takes place in **6**→**7. 8** is a dimer of **7** in which the Sn_2_I_4_ units are opposed to each other. The phosphorous butterfly in the red circle can be opened by breaking bond 1 or 2. **9** points out that the addition of **7** can be repeated. The P‐SnIP helix illustrates the corresponding part of the crystal structure of SnIP comparable to **9**. *P*‐SnIP represents a right‐handed double‐helix strand.

With this important information taken into account, the mechanisms for phosphorene and SnIP can now be deduced with P_4_ (**1**) and SnI_2_ (**2**) as starting materials (Figure 3).

It should be noted that the suggested two mechanisms are in accordance with our experimental findings, but other energetically favorable ones may exist. All molecules discussed in the following mechanisms are fully optimized and converged structures which represent a local minimum on their energy landscapes.

First, we investigate the formation of SnIP from equimolar ratios of the starting materials 3Sn/SnI_4_/P_4_. Starting with the main components P_4_ (**1**), SnI_2_ (**2**) and neglecting the minor ones SnI_4_ (**3**) and I_2_ (**4**), we identified a dimerization of two SnI_2_ molecules to Sn_2_I_4_ (**5**) as the first reaction step. A direct reaction of **1** with **2** as well as the dimer molecule **5** is investigated and leads to two different reaction pathways (Figures 3 and 5, see below). Unfortunately, no thermodynamic data are available for **5** to compare it to all other species in the partial pressure diagrams (Figure 2).

The proposed SnIP mechanism starts with the dimerization of **2** to **5**, which arranges itself in proximity to **1** (cf. Figure 3). Together with a second molecule of **5** the next local minimum structure 2Sn_2_I_4_@P_4_ (**6**) forms. In **6**, first Sn−I bond formation takes place towards [SnI]^+^ chains as two **5** approach each other in a way that leads to alternating Sn/I atoms along the dashed lines in **6**. The coordination of **1** with two entities of **5** to **6** is electronically favored by Δ*E*_tot_=−208 kJ mol^−1^. In this step, due to entropy reasons, the high availability of SnI_2_ in the gas phase and taking kinetic considerations into account, the formation of dimer **5** is favored. It must be stated at this point that we also performed calculations with SnI_2_ monomers instead of **5** dimers coordinating **1**, but such models led to P_black_ formation (see below). Only with a dimer formation of two molecules of **2** to **5** we succeeded in reaching the desired final product SnIP.

In Sn_4_I_4_@P_4_ (**7**) the elimination of four iodine atoms from **6** initiates a structural rearrangement in the outer sphere combined with a P−P bond opening towards a butterfly arrangement in the P_4_ molecule (**1**). This is the energetically most demanding step in the proposed reaction mechanism, with Δ*E*_tot_=+324 kJ mol^−1^.

**Figure 4:**
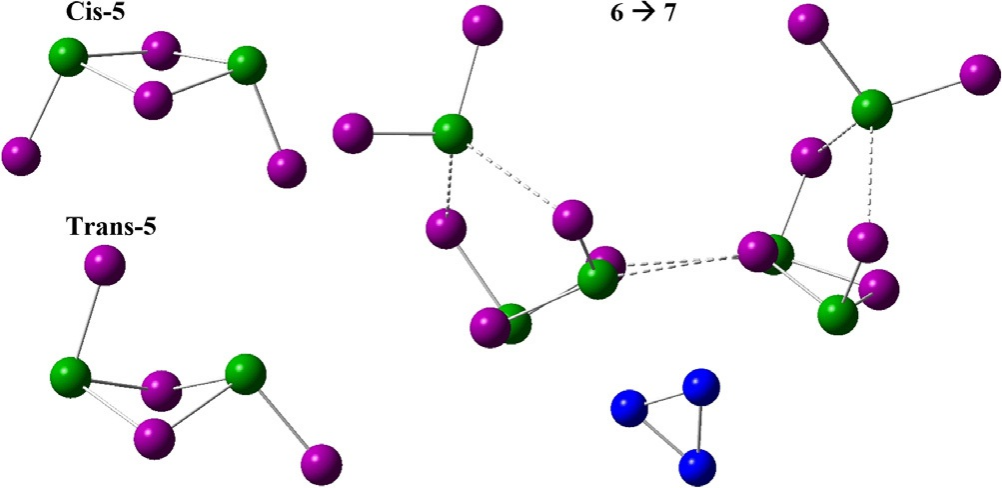
Sn_2_I_4_ molecules (**5**) in *cis* and *trans* configuration. Reaction Scheme for a **6**→**7** reaction pathway. Two SnI_2_ (**2**) attract two *cis*‐**5** entities in 2Sn_2_I_4_@P_4_ (**6**) during bond formation in the Sn−I substructure. In a next step, SnI_4_ (**3**) will emerge during Sn_4_I_4_@P_4_ (**7**) formation. I (purple), Sn (green), and P (blue) spheres. A P atom of P_4_ is covered.

**5** (Δ*E*_tot_=−83 kJ mol^−1^) can be obtained as *cis* or *trans* isomer (cf. Figure 4). The *cis*/*trans* isomerism and abstraction of iodine atoms, which is essential for achieving the final ratio (Sn/I/P=1:1:1), needs further attention. We identified the *cis* isomer of **5** as the most favorable species to coordinate with **1** as stated in Figure 3, step **6**. The preference of the *cis* over the *trans* isomer is due to the following reasons: a) the steric hindrance during formation of **6** by two **5** is much lower; and b) the two lone pairs of **2** can attract two iodine atoms in *cis*‐**5** in such a way that a six‐membered local minimum state is formed. This process allows effective abstraction of four iodine atoms in the Sn−I substructure of **6** by two molecules of **2** (see Figure 4).

Following such a reaction pathway, the formation of iodine (**4**) is avoided, which is consistent with our observation during synthesis [cf. Supporting Information Note 1 and Eq. 1].

(1) $$2\;\left(\mathrm{Sn}{}_{2}I{}_{4}\right)@P{}_{4}\;\left(6\right)+2\;\mathrm{SnI}{}_{2}\;\left(2\right)\to \mathrm{Sn}{}_{4}I{}_{4}@P{}_{4}\;\left(7\right)+2\;\mathrm{SnI}{}_{4}\;\left(3\right)$$

Another, less‐likely **6**→**7** reaction pathway step may involve the intermediate formation of iodine (**4**). Taking this assumption into account, additional **2** is not needed and **4** is formed simultaneously during formation of **7**. We assume that **4** must instantaneously be consumed by an oxidation reaction of elemental Sn to SnI_2_ according to Equation 2.

(2) $$\mathrm{Sn}+I{}_{2}\;\left(4\right)\to \mathrm{SnI}{}_{2}\;\left(2\right)$$

Dependent on the kinetics of such a reaction, an intermediate formation of **4** should lead to visible amounts of **4** during synthesis, which is obviously not the case.

Another observation may help to substantiate the proposed first **6**→**7** reaction pathway mechanism. As stated earlier in this manuscript, Sn and SnI_4_ are used as starting materials for the synthesis of SnIP. It was also reported that SnI_2_ will be formed in a slow comproportionation reaction with a reasonable reaction rate above 633 K.^[19]^ This temperature is only slightly lower than *T*_max_=673 K applied in the SnIP synthesis (see Figure 2). If an equimolar 2Sn/2SnI_2_/P_4_ mixture of starting materials is used instead of 3Sn/SnI_4_/P_4_ where SnI_2_ (**2**) is available directly, one can observe an optimized formation of SnIP. Much larger crystals and less starting material residues occur.^[11a]^ The beneficial usage of preformed SnI_2_ substantiates our proposed mechanism as shown in Figures 3 and 4 and underlines the importance of formation or presence of **2** during the SnIP synthesis.

To complete SnIP formation, two molecules of **7** need to arrange in such a way that two P_4_ butterflies are in close proximity, enabling bond formation in the phosphorus substructure. Bond formation in the [SnI]^+^ substructure and in the [P]^−^ substructure during step **7**→**8** are energetically less demanding processes than the P_4_ activation process in step **6**→**7**. We gain 103.3 kJ mol^−1^ compared to the aforementioned reaction step. A fully open P chain fragment is formed during this reaction step in Sn_8_I_8_@P_8_ (**8**) enabling the inner P helix formation. In a quasi‐polymerization reaction another molecule **7** reacts with **8** to larger units.

SnIP consists of two enantiomeric double helical forms, a left‐handed *M* and a right‐handed *P* double helix.^[20]^ The stereochemistry of the inner [P]^−^ chain is determined in **8**, dependent on the way the butterfly P_4_ ring is opened. In **8** the three‐bonded P atom (marked with an arrow in Figure 3) is attached to two other P atoms in the butterfly. Dependent on which bond (1 or 2, marked by scissors in Figure 3) is broken, a *M* or *P* double helix results, respectively. The outer [SnI]^+^ helix seems to follow the presetting of the inner helix. This suggested mechanism can explain the racemic nature present in the final product where equal amounts of *M* and *P* helices are present.^[11b]^

Finally, the double helix formation starts in **9**, optimizing the overall bonding situation towards the favored double helix arrangement in the final product (the final structure is given in Figure 1). A total energy Scheme is illustrated in Figure 6. Overall, the formation of SnIP is favored by Δ*E*_tot_=−26 kJ mol^−1^ related to the starting materials.

Additional information and DFT data concerning all local minima structures **5** to **9** are summarized in Supporting Information Note 2.

**Figure 5:**
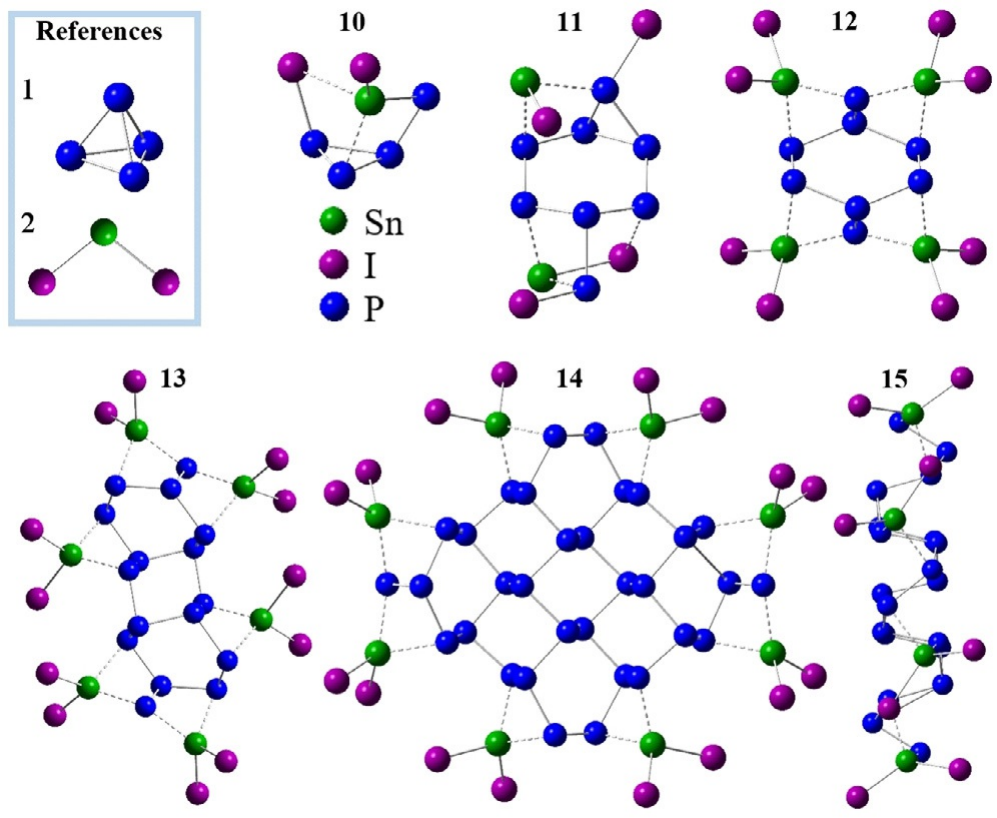
Starting materials (**1**, **2**) and geometry‐optimized structures **10** to **15** in the proposed phosphorene mechanism. **14** and **15** are two different views on the same structure. In **14**/**15** four SnI_2_ coordinate with two‐bonded phosphorus in a highly symmetric way.

In general, the P_black_ synthesis is performed at higher temperatures than the SnIP one. The phosphorene formation (single sheet formation) starts with the activation of **1** by a single SnI_2_ (**2**) molecule (cf. Figure 5). When a tin atom first inserts into one of the P−P bonds, an iodine atom attaches to a neighboring phosphorus atom. Subsequently, **10** consists of two triangles above each other, an almost unchanged P_3_ unit and a Sn‐I‐P unit with the remaining residual iodide coordinated to the tin atom. This local minimum **10** represents the highest energy point on the entire reaction pathway (+76 kJ mol^−1^). **10** can react with another molecule **2** forming a highly symmetric 2(SnI_2_)P_4_ local minimum structure (not shown in Figure 5), which is not able to dimerize further. In **11** the first P−P bonds are formed while SnI_2_ is fragmented totally in [SnI]^+^ and I^−^.

After this first dimerization step, **11** is stabilized by the addition of two additional SnI_2_ molecules (**2**), yielding **12**. With this step, we created a primary essential building block for the phosphorene sheet, the corrugated six‐membered ring in a chair conformation. On all ridges, single‐bonded P atoms with a formal charge of 2− are coordinated by two Sn from both sides while two‐bonded P are mono‐coordinated with one **2**. Homo‐atomically three‐bonded phosphorus atoms exactly represent the bonding situation in an ideal phosphorene sheet. Following the same coordination and bond formation principle, two molecules of **12** can be merged to form **13**. Growth of the phosphorene sheet can now happen in any direction by adding additional **12** molecules to **13** or dimerization of **13**. In each case mentioned so far, SnI_2_ (**2**) is replaced at the ridges where P−P bond formation occurs. For expample, in **14**, a dimerization of **13** took place and four **2** were removed. **15** represents a side view of **14** where the conformation of the P_black_‐related phosphorene layer becomes evident. The growth of a large‐area phosphorene sheet might be possible without bulk P_black_ formation if the attachment of multiple phosphorene sheets is hindered by entities weakly coordinating the remaining lone pairs.

**Figure 6:**
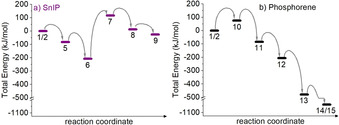
Consecutive reaction steps on the corresponding total energy scale (0 K) for a) SnIP and b) phosphorene. The numbering links each step to the structures in Figures 3 and 4. The arrows are used to clarify the succession and are not related to the actual barriers between the steps.

According to our 0 K calculations the formation of P_black_ (composed by phosphorene layers) is strongly favored (Δ*E*(**14**)=−1040 kJ mol^−1^) and a comparatively small energy portion is needed to form **10** from **1**/**2**. A full total energy Scheme is given in Figure 6 and DFT data are summarized in Supporting Information Note 2.

In both reaction cascades, the most energy‐demanding step is to activate and break the first P−P bond in the P_4_ tetrahedron. This step occurs at different points in the reaction cascade, in step **6** to **7** for SnIP and during step **1**/**2** to **10** in the case of P_black_. The most obvious difference for both mechanisms is the initiation of a P−P bond activation. In the SnIP case, two dimerized Sn_2_I_4_ molecules are needed to coordinate the P_4_ tetrahedron, followed by a concurrent bond opening and double iodide elimination [cf. Eq. (2)] to realize the reactive butterfly P_4_ configuration. The dimerization is energetically favored in our calculations by approx. 200 kJ mol^−1^ (**1**/**2** to **6**). For phosphorene formation at higher temperatures (cooling gradient applied from 923 to 823 K during P_black_ synthesis)^[3b]^ the P_4_ activation (Δ*E*_tot_=+76 kJ mol^−1^, from **1**/**2** to **10**) occurs via a monomeric SnI_2_ molecule (**2**) and by direct insertion into a P−P bond.

While the SnI_2_ (**2**) partial pressure at 673 K, being the synthesis temperature of SnIP (see Figure 2), is comparable to the partial pressure for the P_black_ case (ca. 10^−3^ atm), the dimer formation of **5** should be favored at this temperature and disfavored at higher P_black_ synthesis temperatures.

The role of iodine and metal halides for the formation of phosphorene/P_black_ was subject to intensive research in the past few years.^[21]^ Effective bulk P_black_ formation was observed if tin and iodine species were present as starting materials independent of the source. For almost any synthesis in which SnI_2_ formation from a metal iodide and tin in elemental or intermetallic form was possible, the authors observed P_black_ formation. Besides SnI_2_, the heavier homologue PbI_2_ can act as a less active species for P_black_ formation, but it has a high activity and selectivity in the formation of black arsenic–phosphorus As_0.83_P_0.17_.^[22]^ As_0.83_P_0.17_ crystallizes in the P_black_ structure type.

To elucidate these reaction mechanisms in more detail and to address a situation as close as possible to conditions during synthesis, we performed a DFT analysis at different temperatures and pressures.

**Figure 7:**
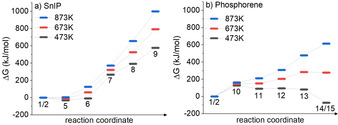
Reaction pathways with Δ*G*(*p*,*T*) for a) SnIP and b) phosphorene calculated at three different temperatures (*T*=473 K, 573 K, or 673 K). In all cases, pressure was set to 50 atm and Gibbs energies were referenced to the starting materials **1** and **2**. The lines between the local minima are drawn to guide the eyes and DFT data are summarized in Supporting Information Note 5.

From Barlow's law, the bursting pressure of our silica glass ampoules was deduced to be 100 bar (ca. 99 atm), but with the safety factor of 1.5, a maximum working pressure of 66 atm can be hypothesized (cf. Supporting Information Note 3).^[23]^ It is also known that the formation takes place during slow cooling starting from maximum temperature for both compounds. Following this train of thought, the assumption of 50 atm seems reasonable while looking at different temperatures. Gibbs free energies (*G*) were calculated for every step mentioned above Figure 6 and the results for Δ*G*(*p*,*T*) with *p*=50 atm are summarized in Figure 7.

We want to point out that for both pathways entropy (calculated by the partition function according Supporting Information Note 4) becomes more dominant the further one moves along the reaction coordinate.

In case of SnIP (Figure 7 a), the dimer formation of **5** is still slightly exothermic at 673 K, which represents the maximum temperature in the synthesis process. Obviously, the dimerization of SnI_2_ (**2**) to Sn_2_I_4_ (**5**) is a spontaneous process and thermodynamically favored at synthesis conditions. At higher temperatures (approaching P_black_ synthesis conditions) Δ*G* becomes positive. Even the attachment of **5** to P_4_ (**1**) shows negative Δ*G* values at low temperatures, which indicates a certain driving force towards the dimer‐driven activation of **1**. After the activation and bond breaking in **1**, each following step shows positive Gibb's free enthalpies up to significant, high values at 873 K; but at the lowest examined temperature of 473 K the Gibb's free enthalpy is halved from the one at 873 K, which indicates a more feasible formation tendency at lower temperatures. This feature is consistent with our observations that SnIP cannot be prepared at higher temperatures than 673 K. It needs to be stated at this point that SnIP decomposes peritectically at 740 K to Sn_24_P_19.3_I_8_, which is the thermodynamically stable phase under these conditions. Therefore, at 873 K all SnIP data need to be interpreted with care or, in other words, it is most likely that SnIP cannot be formed at such high temperatures. Going back to the synthesis of SnIP, the starting materials are cooled down from 673 K with a rate of 5 K h^−1^, which means the optimal reaction conditions are met somewhere between 673 K (Figure 7 a, red lines) and 473 K (Figure 7 a, black lines) from an experimental point of view. The calculated free energies for the formation of **9** are 576 kJ mol^−1^ at 473 K and 793 kJ mol^−1^ at 673 K. In a previous work the electronic energy contribution at 0 K for seven agglomerating SnIP double helices was found to be 879 kJ mol^−1^ already.^[12b]^ Therefore, one can expect that lattice energy can compensate the positive formation energies calculated in this study.

The Gibbs free energy profile for the phosphorene formation mechanism across the different reaction steps is different in the case of P_black_ (Figure 7 b). The first P_4_ activation step **1**/**2** to **10** is the crucial one in the entire reaction cascade. This finding is equivalent to the previously discussed SnIP mechanism, where the dimerization of two SnI_2_ molecules is favored at low temperatures in the vicinity of **1** during P−P bond activation. Obviously, the P−P bond activation and insertion of a single SnI_2_ molecule into a P−P bond is the most energy‐demanding step at elevated temperatures. This feature, taken from our 0 K calculations, is also valid at 473 K. If the temperature is raised further the following steps in the reaction cascade become more and more endergonic. At 673 K we derived a Δ*G* of 278 kJ mol^−1^ and at 873 K of 613 kJ mol^−1^ from our DFT data for the last reaction step **14**/**15**. A huge lattice energy contribution is needed to form P_black_ in the end. This seems to be the case because bulk P_black_ is formed in a temperature interval of 923 to 823 K upon slow cooling.^[3b]^ We see a clear trend towards less positive and even negative Δ*G* upon reducing the temperature in our calculations. Somewhere between 673 and 473 K Δ*G* becomes negative for the last reaction step. This trend can perfectly explain the effective and rapid growth rate of P_black_ found between 773 and 673 K (and applying a temperature cooling rate of 100 K h^−1^) in an in situ neutron diffraction experiment,^[3b]^ in particular if the lattice energy gain during crystallization is also taken into account. Additional information concerning the in situ neutron experiment is given in the Supporting Information.

## Conclusion

The formation mechanisms of phosphorene, a monolayer unit of P_black_, and SnIP, a double helical inorganic semiconductor, were investigated in the framework of DFT. Both compounds can be prepared at different temperatures by a gas phase reaction with phosphorus, Sn, and SnI_4_ as starting materials. While bulk P_black_ is formed at elevated temperatures and quasi‐catalytic amounts of SnI_2_, the formation of SnIP takes place at lower temperatures and equimolar ratios of all necessary elements. In a first step, CalPhaD methods were successfully used to identify the main gas phase species during both syntheses: P_4_ and SnI_2_. The latter is formed by a comproportionation reaction of Sn and SnI_4_ above 633 K and represents the active gas phase species in both reactions. Iodine formation was fully ruled out by in situ observation during synthesis and our CalPhaD results.

Both reaction mechanisms were illustrated by total energy calculations at 0 K with geometry‐optimized local minimum structures. In each case, P_4_ activation is the most energy demanding reaction step. A Sn_2_I_4_ dimer is crucial in the SnIP reaction cascade to activate the P_4_ molecule while a direct insertion of SnI_2_ into a P−P bond of P_4_ takes place during P_black_ synthesis. Low temperatures and high SnI_2_ availability (due to the equimolar ratio of starting materials) in the SnIP case allows effective Sn_2_I_4_ dimer formation whereas such dimers are not stable (and present) at higher temperatures during P_black_ formation. As a direct consequence, the beneficial usage of SnI_2_ as a starting material for optimized SnIP synthesis becomes obvious. Also, the previously reported favorable P_black_ synthesis in presence of Sn/I sources is a straightforward result of our proposed mechanism. In each case where catalytic amounts of SnI_2_ can be realized during synthesis, an effective formation of P_black_ was found.

Gibbs free energy calculations were performed at elevated temperatures of 473, 673, and 873 K and a pressure of 50 atm in order to gain substantial information about the two reaction processes as close as possible to the applied synthesis conditions. Even at the SnIP synthesis temperature of 673 K the Sn_2_I_4_ dimer formation is energetically and thermodynamically favored, which corroborates our 0 K calculations. While all SnIP reaction steps are energetically demanding along the reaction pathway, the situation is slightly different for phosphorene. The more the temperature is lowered the more favorable the formation of phosphorene becomes. At the lowest temperature in our calculations, at 473 K, the growth of phosphorene in lateral dimensions shows negative Δ*G* values, which illustrate the thermodynamic stability of phosphorene sheets under these conditions. A very fast growth of P_black_ observed during a neutron diffraction experiment between 773 and 673 K—more than 150 K lower than the synthesis temperature itself—can perfectly be explained by our results.

CVD processes are an important technique to deposit material in thin films. With the knowledge of the formation mechanisms for SnIP, P_black_, and phosphorene, such important one‐ and two‐dimensional materials might be effectively deposited on substrates in the future. Based on this knowledge, CVD processes can be adjusted and optimized aiming for crystalline and maybe oriented materials on various substrates.

## Conflict of interest

The authors declare no conflict of interest.

## Supporting information

As a service to our authors and readers, this journal provides supporting information supplied by the authors. Such materials are peer reviewed and may be re‐organized for online delivery, but are not copy‐edited or typeset. Technical support issues arising from supporting information (other than missing files) should be addressed to the authors.

Supplementary

Supplementary

Supplementary

Supplementary

Supplementary
