# Supplementary material for: Formation Mechanisms for Phosphorene and SnIP
Source: Angew Chem Int Ed Engl. 2021 Feb 15;60(12):6816–23. doi: 10.1002/anie.202016257 (PMC7986658; doi:10.1002/anie.202016257)
Supplement: Supplementary file 1 — Supplementary [file ANIE-60-6816-s004.pdf]

Angewandte Chemie
Eine Zeitschrift der Gesellschaft Deutscher Chemiker

Supporting Information

Formation Mechanisms for Phosphorene and SnIP

*Markus R. P. Pielmeier and Tom Nilges**

anie_202016257_sm_miscellaneous_information.pdf
anie_202016257_sm_Pblack923.mp4
anie_202016257_sm_SnIP673.mp4

Supplement

*

Computational Methods [14]

Ab initio calculations are carried out using the Gaussian09 code and its default settings unless noted otherwise. All optimizations and frequency calculations are performed on the DFT-GGA-level with the functional from Perdew, Burke and Ernzerhof (PBE). For basis sets the Gaussian09 integrated SDD setting was chosen. This means for phosphorus the Dunning full double zeta basis set[36] was used, while for tin and iodine polarizable effective core pseudopotential (ECP) basis sets[37] were applied. The thermodynamic data was generated with the Gaussian09 thermochemistry analysis tool starting from the corresponding local minimum. All relevant data are deposited as files (cf. Note 2 and Note 5)

Structure models illustrated in this paper were generated with the GaussView5 visualization tool. The default value of 75% of vdW radii is shown on all figures for all atoms except in wireframe representation.

The gas phase composition was calculated with the tragmin5.1 code and its default thermodynamic constants in the respective temperature regimes.[15] 3 solid phases derived from our

experiments and 5 gas phase species were used as input parameters. More detailed data and parameters concerning the gas phase evaluations are summarized in the Supplement Note 5.

Black phosphorus and SnIP Syntheses

Despite obvious structural differences, the synthesis procedures for both compounds are rather similar. In both cases tin, Sn-(IV)-iodide and amorphous red phosphorus (P_{red}) act as starting materials. In the case of P_{black} catalytic amounts of Sn and SnI_4 are used to form P_{black} from P_{red} via a short way transport reaction. Bulk P_{black} can be grown in a temperature range from 923 K to 823 K with a cooling rate of 13.3 K/h.[3b] In an in situ neutron diffraction experiment Pblack showed a reasonable growth rate even at lower temperatures. Within 75 minutes, P_{black} grew to large crystals with edge lengths up to 0.5 cm in evacuated silica ampoules.[3b] During this experiment, a temperature window of 773 to 673 K and using a temperature cooling rate of 100 K/h. For SnIP, equimolar ratios of the elements are used instead, the reaction temperature is lowered to 673 K, and slower cooling with a gradient of 5 K/h is applied. These similarities inspired us to investigate the formation mechanism for both compounds in more detail.

SnIP crystallizes monoclinically, in space group P2/c (No. 13). It contains Z=14 formula units in the unit cell, with lattice parameters of a = 7.934 Å, b = 9.802 Å, c = 18.439 Å and β = 110.06°.[10]

In 2007, Lange et al. reported on the synthesis of P_{black} and identified the main gas phase species present in the reaction vessel that are P_4 (I) and SnI_2 (II).[16] Minor gas phase components like P_2, SnI_4 (III) and I_2 (IV) were also taken into account, stated here in decreasing order of appearance. No significant impurities like halide containing species were found in the final product which lead to the conclusion that no tin or iodine is incorporated in the final product. Any tin or iodine species

can therefore be present only in the gas phase directing the formation of P_{black} during the short way transport process. In the past few years, several studies were performed to identify a possible reaction mechanism for this P_{red} to P_{black} gas phase transformation reaction in CVD processes.[10a] In solution a reaction mechanism has been evaluated and investigated, featuring a nucleophilic attack of a free lone pair of ethylenediamine (en) to P_{white} (P_4 molecule)[7a] and P_{red}.[10b] Here, the P_4 entity (P_{white}) is opened to a reactive species which tends to rearrange into layers of corrugated P_{black} sheets afterwards. Unfortunately, this solution based synthesis with ethylenediamine leads to P-N bond formation and Nitrogen impurities in P_{black} which has been proven by XPS.[10b] Such impurities which come along in addition to common oxygen impurities caused by oxidation processes in solution and during workup procedures can affect the performance of P_{black} in applications. Using a gas phase-based synthesis route, the solvent influence can fully be suppressed and the oxidation problem can be minimized. Therefore, the gas phase-based synthesis remains a crucial method to grow pure, highly crystalline, large area crystals of P_{black} which then can be used for a top-down fabrication of phosphorene.

A detailed mechanism for the gas phase-based P_{black} synthesis which includes a quantum chemical investigation with local minima structures along the reaction pathway or alternatively the identification of any intermediate species on the reaction pathway are lacking. So far, only assumptions, based on observations during the synthesis, like the occurrence of epitaxial growth of P_{black} on $Sn_{24}Sn_{19.3}I_8$ or the formation of other phosphorus allotropes as precursor states are used to explain or interpret the formation of P_{black}. Two full reaction schemes based on the information from Figure 6 are shown in Figure S1 and S2.

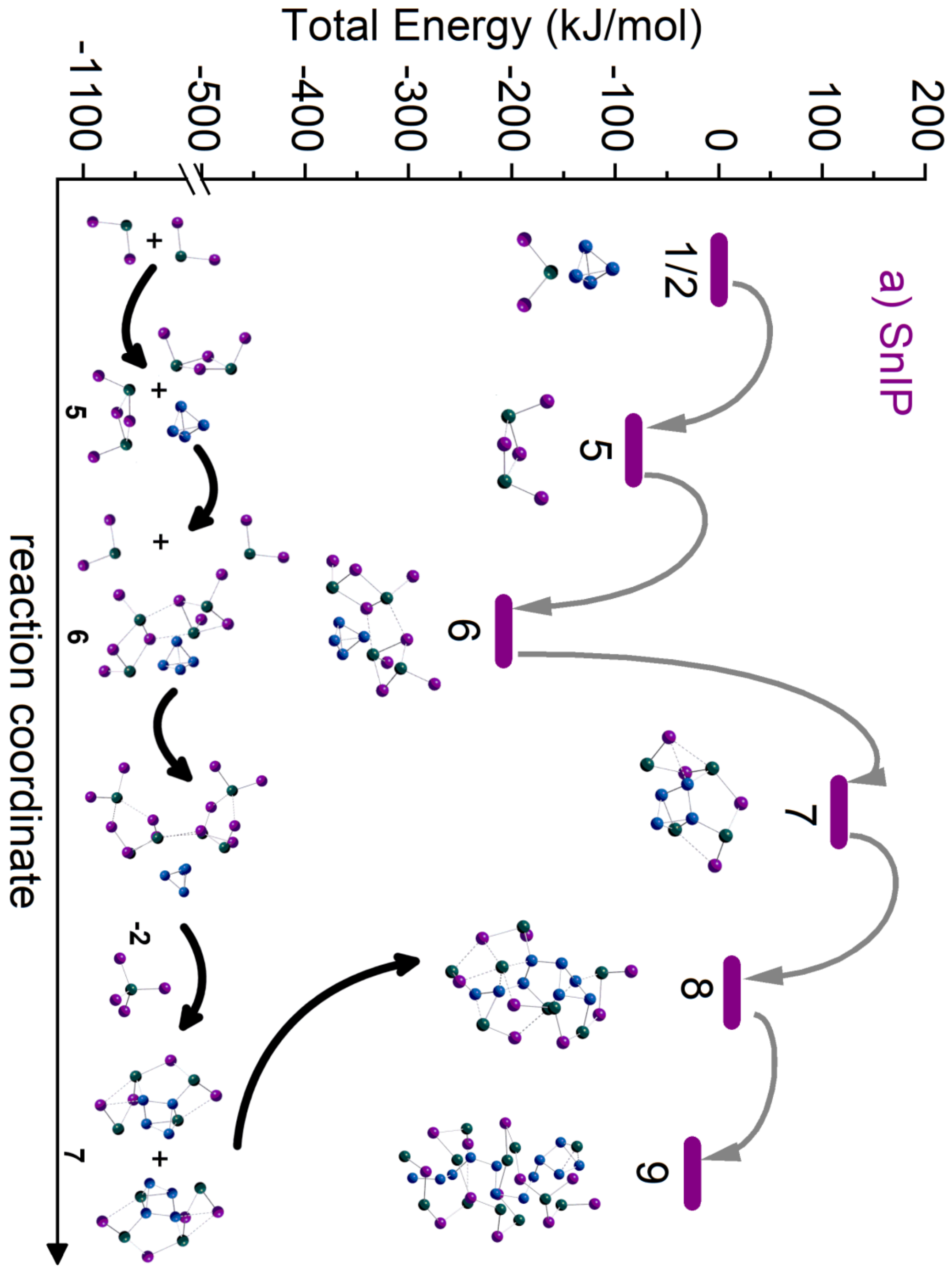

Figure S1. Formation mechanism of SnIP based on total energy calculations (Figure 6). Local mimimum structures are denoted for each step. A rection scheme is shown in the bottom part of the figure for clarity.

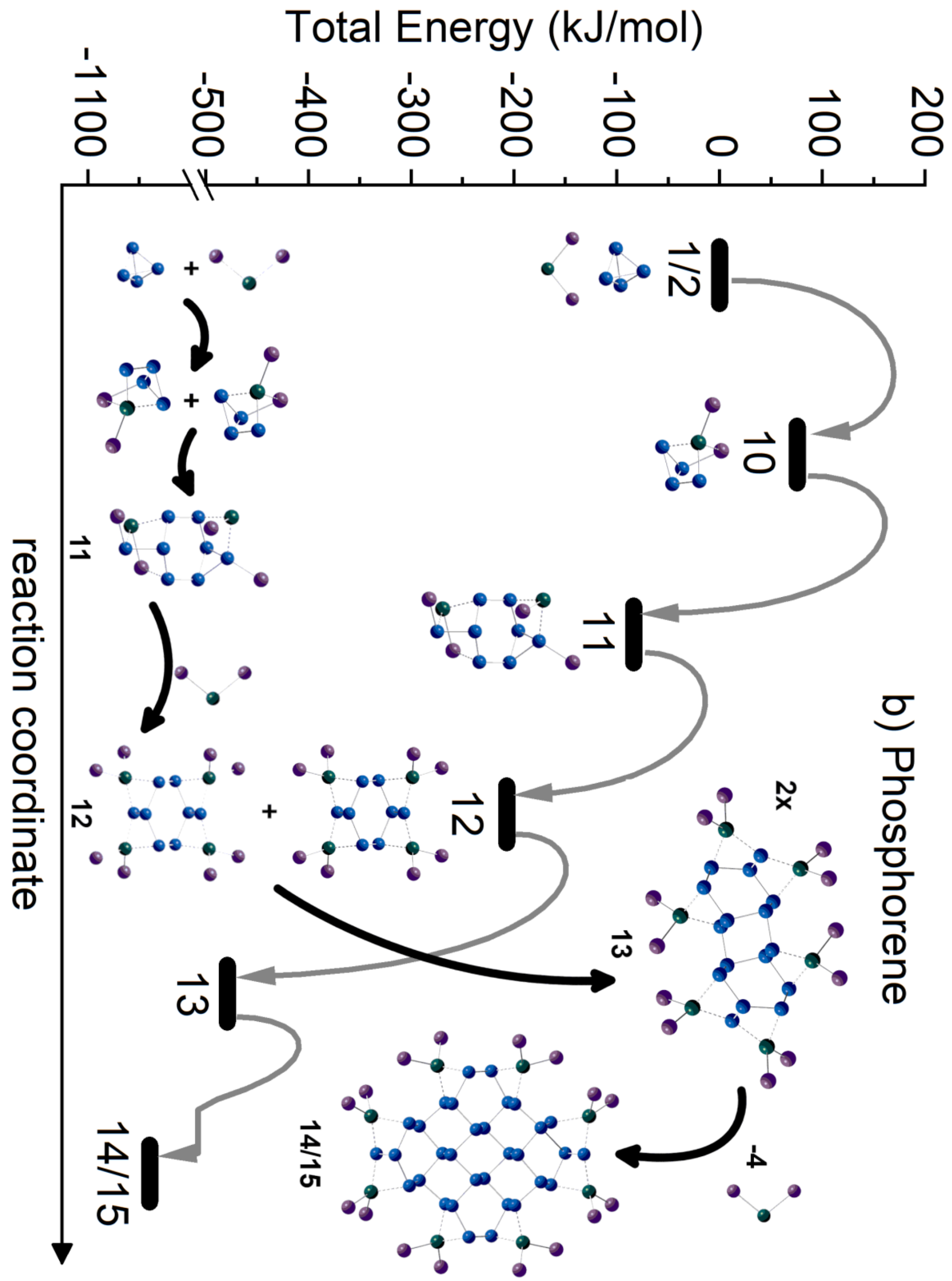

Figure S2. Formation mechanism of P_{black}/phopshorene based on total energy calculations (Figure 6). Local mimimum structures are denoted for each step. A rection scheme is shown in the bottom part of the figure for clarity.

In situ neutron diffraction study on black phosphorus

The formation of formation of black phosphorus was followed in situ in a Neutron diffraction experiment at the ILL, Grenoble, D20 beamline (take off angle 118°; λ = 1.87 Å; cooling rate 100 K/h, PSD detector; data acquisition time 5 min.[3b] All relevant Neutron data are summarized in Figure S3 and additional experimental data or phase analysis results can be taken from literature.[3b] We found only reflections from black phosphorus in our neutron diffraction experiment and indexed the reflections according structure data[16] from the literature. The evolution of the five strongest P_{black} reflections (040), (151), (023)/(240), (061)/(132), and (261)/(172) are denoted in Figure S3. We found no other crystalline phases than black phosphorus and observed a fast growth within minutes during cooling from 773 K.

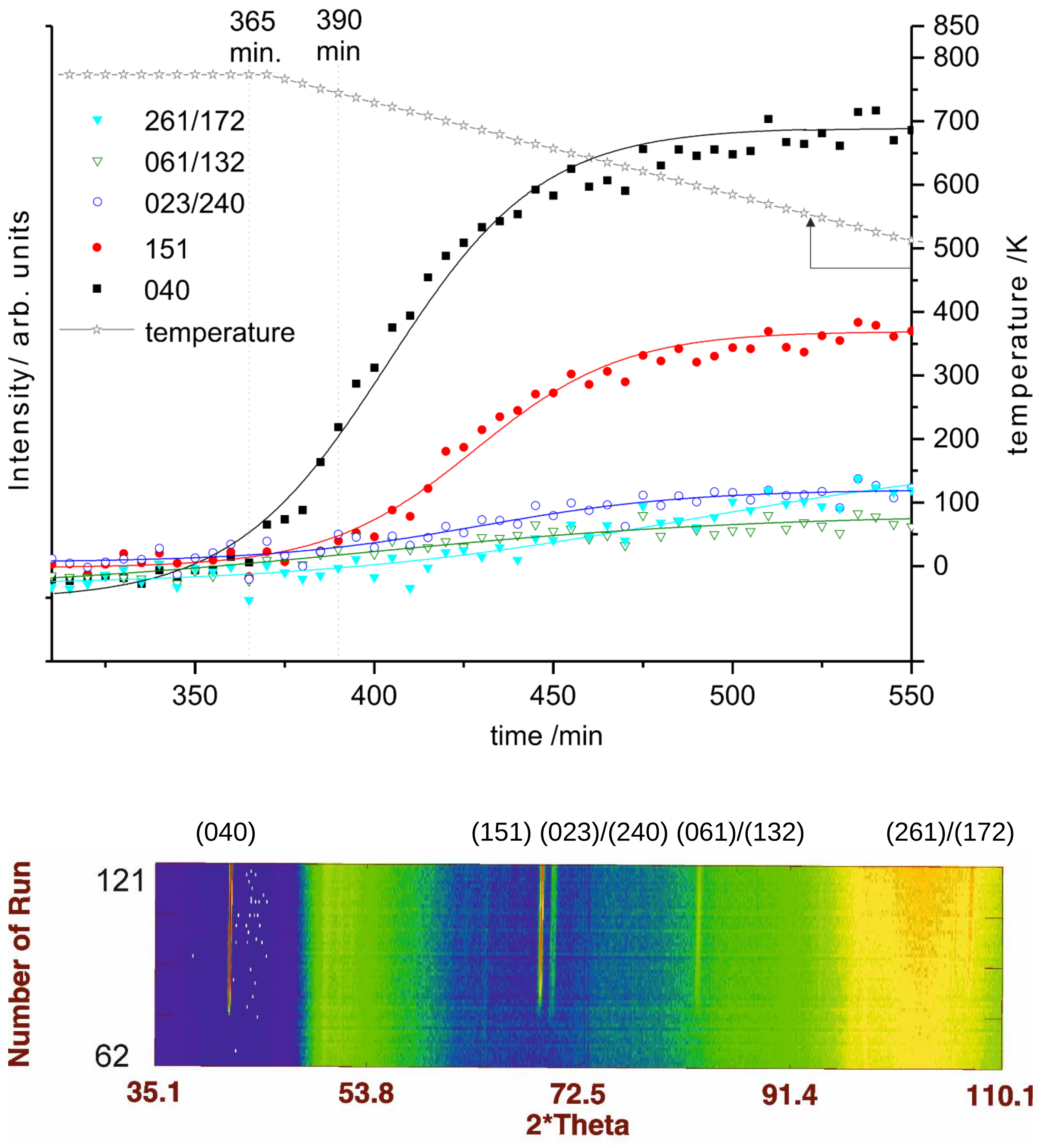

Figure S3. In situ Neutron diffraction study on black phosphorus formation. Data are collected at ILL, Grenoble, D20 beamline. We observed fast growth of P_{black} within approx. 100 min from 350 to 450 minutes of total experiment time. Intensities of five observed P_{black} reflections, equivalent to run 72-121 in the lower section, were observed during cooling.

Note 1 - Quasi in situ observation of the synthesis processes

The videos were taken at synthesis temperature (T) directly after the oven was opened. The name of the deposited file indicates the target compound (SnIP or P_{black}) in the ampoules, synthesis temperature T and the file format (.mp4). A dark red condensation product can be noticed on the walls of the P_{black} ampoule while the atmosphere is orange. When opening the oven during SnIP synthesis, there is no obvious condensation process visible on the walls of the ampoule and an orange atmosphere comparable with the one in the P_{black} synthesis is present. Behind the SnIP ampoule shown in the SnIP673K.mp4 file we placed three ampoules containing 25, 50 and 100 mg of iodine (**4**) in order to illustrate the occurrence of significant amounts of iodine in the gas phase. In each iodine ampoule a dark violet/black gas phase is formed which is in significant contrast to the orange gas phase during the P_{black} and SnIP syntheses.

Pblack923K.mp4; SnIP673K.mp4

We therefore conclude that elemental iodine is not present in significant amounts during both syntheses.

Note 2 - Gaussian data sets of DFT calculations

All data sets containing relevant structure data and additional crucial information are deposited in SnIP.zip and Pblack.zip . The names of all files contain a roman numeral (RN) which represents the local minimum structures respective according to Figures 3 and 5. For local minima structure optimization (at 0 K) there is no further extension in the file name. In the case of Gibbs Free Energy calculations, the pressure was set to 50 atm and the respective temperature (T) was added to the name. For example:

RN = II, no T → II.log

RN = II, T = 473 K → II_473K.log

Structures can be visualized directly from the data via the GaussView visualization package. Interested readers can access or download the data directly from the journal.

Note 3 - Barlow's Formula

Barlow's Formula is used to estimate the bursting pressure of a silica glass ampoule.[23a] The pressure ***P*** represents the estimated maximum internal pressure for a specified tube. ***d*** and ***t*** are the diameter and wall thickness. ***s*** illustrates the tensile strengths which was chosen from the material specifications of Heraeus.[23b] The safety factor ***f*** is used to ensure a safe working environment in the laboratory.

$$P = 2\frac{t \cdot s}{d \cdot f} \approx 66\ bar$$

$$d = 0.01\,m; t = 0.001\,m;\ s = 5 \cdot 10^7\ {}^{N}/_{m^2}\,;\ f = 1.5$$

Taking our ampoule specifications into account we calculated a bursting pressure of approximately 66 bar. For the DFT calculations we used an internal pressure of 50 bar in the ampoule which is a reasonable estimate of the maximum operation conditions during our syntheses.

Note 4 - Thermodynamic calculations in Gaussian

The starting point for all thermodynamic values is the partition function **q** for a given component. Other variables are temperature (**T**), volume (**V**) and pressure (**p**), while the universal gas constant (**R**) is also needed.

$$S = R + R\ln\big(q(V,T)\big) + RT\ \left(\frac{\delta\ ln\ q}{\delta T}\right)$$

Note 5 - Data summary

In this note we summarize data used in Figures 6 and 7.

Table 1: Energy values in Hartree derived from DFT calculations for all structures at three different temperatures.

	Etot (0 K)	G (473 K)	G (673 K)	G (873 K)
**I**	-1364.648945	-1364.685663	-1364.709047	-1364.734279
**II**	-26.342895	-26.394046	-26.420961	-26.449208
**III**	-49.247987	-	-	-
**IV**	-22.848169	-22.885933	-22.905297	-22.925518
**V**	-52.717277	-52.799683	-52.846602	-52.896570
**VI**	-1470.099776	-1470.264971	-1470.369821	-1470.483381
**VII**	-1424.279940	-1424.388365	-1424.460196	-1424.538444
**VIII**	-2848.643500	-2848.831077	-2848.963887	-2849.109908
**IX**	-4272.982251	-4273.250507	-4273.444899	-4273.659306
**X**	-1390.963026	-1391.031276	-1391.074728	-1391.121753
**XI**	-2782.015460	-2782.124826	-2782.201810	-2782.286313
**XII**	-2834.748296	-2834.911130	-2835.024328	-2835.148469
**XIII**	-5616.835658	-5617.075676	-5617.253815	-5617.450786
**XIV/XV**	-11128.330885	-11128.665072	-11128.934059	-11129.234224

Table 2: Differential total energies ΔEtot and ΔG, referenced to the starting materials. ΔEtot and ΔG values in kJ/mol given for all local minimum structures **V** to **XV**.

	**ΔEtot**	**ΔG (473 K)**	**ΔG (673 K)**	**ΔG (873 K)**
**V**	-82.7	-30.4	-12.3	4.8
**VI**	-208.0	-8.2	60.6	125.3
**VII**	116.1	266.7	320.5	371.8
**VIII**	12.8	390.8	526.9	656.9
**IX**	-25.4	576.0	792.7	999.9
**X**	75.6	127.1	145.1	162.1
**XI**	-83.4	90.8	152.8	211.7
**XII**	-206.9	95.5	203.7	306.9
**XIII**	-479.1	82.0	283.9	476.6
**XIV/XV**	-1039.9	-71.9	278.3	613.4

Tragmin values

The used parameters in all TRAGMIN evaluations were extracted from the database and summarized in the data file following the program routine for data files.

TragminParameters.dat
